# Heterogenous transmission and seroprevalence of SARS-CoV-2 in two demographically diverse populations with low vaccination uptake in Kenya, March and June 2021

**DOI:** 10.12688/gatesopenres.14684.2

**Published:** 2023-10-09

**Authors:** Patrick K. Munywoki, Godfrey Bigogo, Carolyne Nasimiyu, Alice Ouma, George Aol, Clifford O. Oduor, Samuel Rono, Joshua Auko, George O. Agogo, Ruth Njoroge, Dismas Oketch, Dennis Odhiambo, Victor W. Odeyo, Gilbert Kikwai, Clayton Onyango, Bonventure Juma, Elizabeth Hunsperger, Shirley Lidechi, Caroline Apondi Ochieng, Terrence Q. Lo, Peninah Munyua, Amy Herman-Roloff

**Affiliations:** 1 Division for Global Health Protection, Global Health Center, U.S. Centers for Disease Control and Prevention (CDC)-Kenya, Nairobi, Kenya; 2 Centre for Global Health Research, Kenya Medical Research Institute (KEMRI), Kisumu, Kenya; 3 Global Health Program, Washington State University – Global Health Kenya (WSU-GH Kenya), Nairobi, Kenya; 4 Paul G. Allen School of Global Health, Washington State University, Pullman, Washington, USA; 5 Centre for Global Health Research, Kenya Medical Research Institute (KEMRI), Nairobi, Kenya

**Keywords:** Population-based, Households, Serosurvey, Serology, SARS-CoV-2, COVID-19, Rural, urban informal settlement, transmission, seroprevalence, Kenya

## Abstract

**Background:**

SARS-CoV-2 has extensively spread in cities and rural communities, and studies are needed to quantify exposure in the population. We report seroprevalence of SARS-CoV-2 in two well-characterized populations in Kenya at two time points. These data inform the design and delivery of public health mitigation measures.

**Methods:**

Leveraging on existing population based infectious disease surveillance (PBIDS) in two demographically diverse settings, a rural site in western Kenya in Asembo, Siaya County, and an urban informal settlement in Kibera, Nairobi County, we set up a longitudinal cohort of randomly selected households with serial sampling of all consenting household members in March and June/July 2021. Both sites included 1,794 and 1,638 participants in the March and June/July 2021, respectively. Individual seroprevalence of SARS-CoV-2 antibodies was expressed as a percentage of the seropositive among the individuals tested, accounting for household clustering and weighted by the PBIDS age and sex distribution.

**Results:**

Overall weighted individual seroprevalence increased from 56.2% (95%CI: 52.1, 60.2%) in March 2021 to 63.9% (95%CI: 59.5, 68.0%) in June 2021 in Kibera. For Asembo, the seroprevalence almost doubled from 26.0% (95%CI: 22.4, 30.0%) in March 2021 to 48.7% (95%CI: 44.3, 53.2%) in July 2021. Seroprevalence was highly heterogeneous by age and geography in these populations—higher seroprevalence was observed in the urban informal settlement (compared to the rural setting), and children aged <10 years had the lowest seroprevalence in both sites. Only 1.2% and 1.6% of the study participants reported receipt of at least one dose of the COVID-19 vaccine by the second round of serosurvey—none by the first round.

**Conclusions:**

In these two populations, SARS-CoV-2 seroprevalence increased in the first 16 months of the COVID-19 pandemic in Kenya. It is important to prioritize additional mitigation measures, such as vaccine distribution, in crowded and low socioeconomic settings.

## Introduction

The severe acute respiratory syndrome coronavirus 2 (SARS-CoV-2), and the resulting disease, coronavirus disease (COVID-19) was declared a public health emergency of international concern (PHEIC) by the World Health Organization (WHO) on 30 January 2020 ^1, 2^. By the first week of January 2022, close to 300 million confirmed cases and over 5.4 million deaths had been reported worldwide ^3^. In Kenya, since 13 ^th^ March 2020, when the first case of SARS-CoV-2 infection was reported, a total of 295,098 cases and 5,378 deaths (case fatality rate of 1.8%) had been reported by the Ministry of Health (MoH) as of 31 ^st^ December 2021 ^4^. In response to the pandemic, the Kenyan MoH employed various strategies, including advocating for the 3Ws (for instance, wearing of masks, washing your hands, watching distance), restriction of movement, and later administration of COVID-19 vaccine to reduce the risk of infection and severe disease.

SARS-CoV-2 has extensively spread in cities and rural communities. Most infected persons generate detectable antibodies that persist for up to a year ^5^. Leveraging existing surveillance programs for COVID-19 surveillance and for seroprevalence surveys offer an opportunity to accelerate the monitoring of the extent of transmission of SARS-CoV-2 infections in populations. Population-based surveillance platforms have especially been singled out as integral in generating data to show the temporal and spatial spread of the SARS-CoV-2 virus; as well as evaluate the impact of non-pharmaceutical and pharmaceutical interventions ^6^. Furthermore, systematic longitudinal surveillance in these platforms provides the much-needed information on the magnitude of exposure in the population and the duration of immune responses among the infected individuals. These data are useful in informing the MoH and other agencies on the magnitude of population exposure to SARS-CoV-2 infection and in influencing the design and delivery of public health mitigation measures.

To inform the COVID-19 response in Kenya, active (household-based serial longitudinal serosurveys) and passive (health facility screening for patients presenting with respiratory symptoms) surveillance approaches were activated to track the extent of the spread of SARS-CoV-2 infections in two well-defined populations in an urban, densely populated, informal settlement in Nairobi and a rural setting in western Kenya ^7, 8^. We present findings from the longitudinal household-based serial seroprevalence surveys with a detailed context of SARS-CoV-2 circulation in these populations. The seroprevalence surveys were aligned with the UNITY seroepidemiological protocol by WHO ^9^ and followed a serosurvey performed in December 2020 in Kibera, Nairobi ^10^. Describing the SARS-CoV-2 transmission waves against the temporal patterns of population-level seroprevalence further provides much needed data to inform the review, revision, and implementation of context-appropriate mitigation strategies, including the deployment of vaccines.

## Methods

### Study site and population

**Figure 1.**
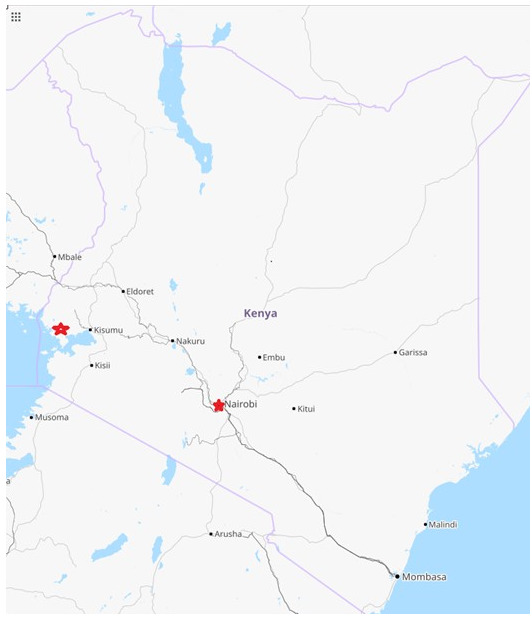
Kenyan map showing population-based infectious disease surveillance (PBIDS) sites (red) in Asembo, Siaya County in western Kenya and Kibera informal settlement, Nairobi County, Kenya. *Maps data: Google, ©2022*.

The study leveraged on an ongoing population-based infectious disease surveillance (PBIDS) platform. The PBIDS platform conducts surveillance within two well-characterized populations in Asembo (rural western Kenya in Siaya County) and in Kibera (the largest urban, densely populated, informal settlement in Nairobi) and is run by Kenya Medical Research Institute-Centre for Global Health Research (KEMRI-CGHR) with technical and financial support from US Centers for Disease Control and prevention (CDC) ^7, 8^. The platform has been running since 2006 and the surveillance objectives and methods have been described previously ^7, 8^. Briefly, the PBIDS platform aims to monitor burden and aetiology of common and (re)emerging acute infectious diseases and evaluate the impact of public health interventions in the two populations in Kenya, Figure 1. In Kibera, PBIDS covers two villages characterized by very poor water purification, supply, and waste disposal, and a large number of infectious diseases including an adult HIV prevalence of 15% ^7, 11^. The surveillance area covers 0.40 km ^2^, with a high population density (~77,000 individuals/km ^2^). The Asembo site covers 33 villages that are sparsely populated (~350 individuals/km ^2^) in ~80 km ^2^. The area is culturally homogeneous (95% Luo ethnicity); subsistence farming and fishing constitute the principal economy. The area has perennial, high-level malaria transmission and has reported high prevalence of HIV (~15%) among adults aged 15–64 years. Consequently, both sites have mortality rates that reflect a high number of infectious diseases ^12– 14^.

Enrolled households are followed regularly (once in 2020 due to COVID-19 interruptions from April to September and three times in 2021) to collect data on recent illnesses, healthcare-seeking behaviour, and demographic characteristics. The households are within 5 km radius of St Elizabeth Lwak Mission Hospital (LMH) in Asembo and a 1 km radius of the Tabitha Medical Clinic in Kibera. The active PBIDS participants receive free medical care for acute illnesses at the centrally located surveillance clinics. All households within the defined radius are invited to participate in PBIDS and enrolment is continuous. As of December 2020, the Asembo PBIDS had 34,999 persons in 9,225 households, while Kibera PBIDS had 23,103 persons in 5,265 households under follow-up.

### Household-based seroprevalence surveys

A longitudinal cohort of randomly selected households from the PBIDS database were enrolled and serial sampling of all available household members conducted in March and June 2021, Figure 2. The target was to enrol approximately 900 individuals in each site. For unavailable households and household members, three attempts for enrolment were made before randomly selected replacement households were considered. The survey size was anticipated to detect a seroprevalence of 45% with a precision of 5% and design effect of 2 at 95% confidence interval. An attrition of 20% was also incorporated. Our expected seroprevalence of 45% was informed by earlier serosurveys reporting a seroprevalence of 13% among blood bank specimens in Kenya ^15, 16^ and an observation of 3.6 times higher seroprevalence among informal settlement residents compared to formal settlement residents in India ^17^.

**Figure 2.**
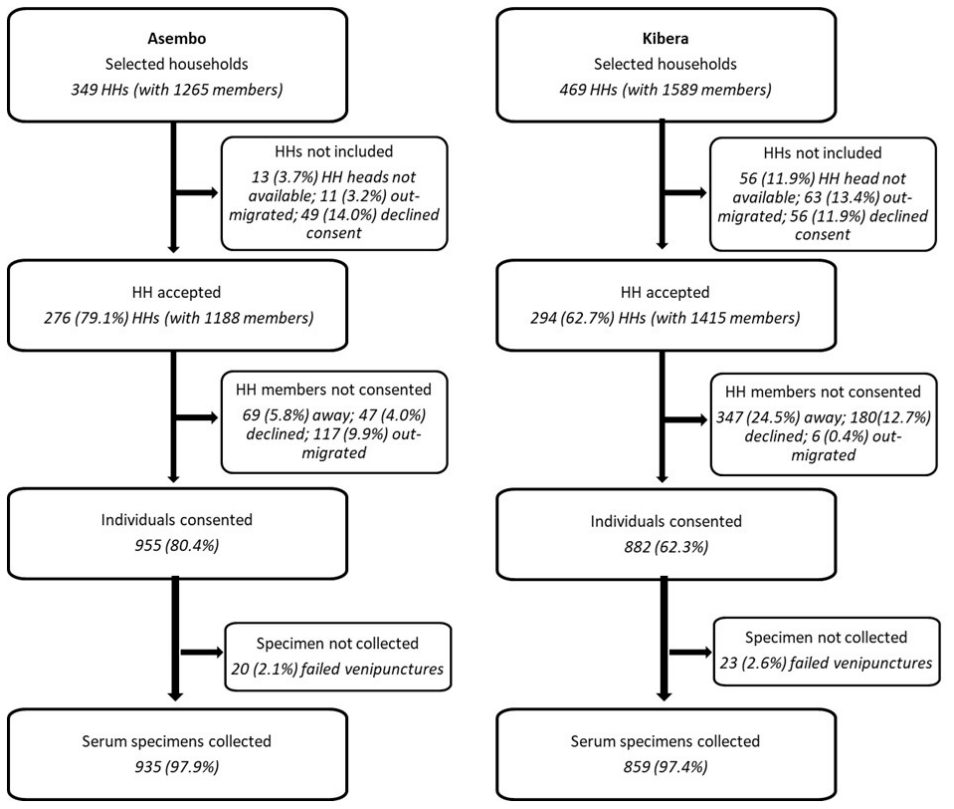
Flow chart showing household (HH) enrolment, participant consenting and specimen collection in Asembo, Siaya County and Kibera, Nairobi County, Kenya.

The household members’ consenting, enrolment, data and blood collection were conducted through home visits by trained field teams consisting of a field worker and a phlebotomist. Venous blood samples (approximately 5 ml for persons aged >12 years; 2–3 ml for children 2–12 years and 1.5 ml for children <2 years) were collected from each participant and transported in a cool box at 2-8°C to the laboratory on the same day. Sera were separated from the whole blood specimen and stored at -80°C before testing.

### COVID-19 surveillance at the PBIDS health facilities

At the surveillance clinic in each site, PBIDS participants of all ages presenting with severe acute respiratory illness (SARI) were consented for a nasopharyngeal and oropharyngeal (NP/OP) specimen. Since 1 ^st^ May 2020, all the NP/OP specimens collected were tested for SARS-CoV-2 using real time Reverse Transcription-Polymerase Chain Reaction (rRT-PCR). From September 2020, the eligibility criterion for NP/OP collection was expanded to include any patients presenting with acute febrile illness (AFI) or acute respiratory illness (ARI) as well as known contacts of confirmed COVID-19 cases regardless of their symptom status. Table 1 presents the case definitions used in the enhanced COVID-19 surveillance.

**Table 1.** Case definitions of the various inclusion criteria used in PBIDS for health facility COVID-19 surveillance in Asembo, Siaya County and Kibera, Nairobi County, Kenya.

Case ^1^	Definition
SARI	*For <5 years:* patients presenting with cough or difficulty breathing, plus chest indrawing or oxygen saturation <90% or WHO danger signs (inability to feed, impaired consciousness, vomiting). *For >=5 years:* patients presenting with cough or difficulty breathing or chest pain, plus temperature >=38.0°C or oxygen saturation <90%.
AFI	Patients presenting with temperature of >=38.0°C or recent history of fever.
ARI	Patients presenting with cough or difficulty in breathing or running/blocked nose, or sore throat.
COVID-19 contacts	Patients reporting close contacts with confirmed COVID-19 cases regardless of their symptom status.

Key: 1, SARI was used for COVID-19 surveillance from May 1 ^st^, 2020 and the rest added from September 1 ^st^, 2020 onwards. PBIDS, population based infectious disease surveillance; COVID-19, coronavirus disease; SARI, Severe acute respiratory illness; AFI, Acute febrile illness; ARI, Acute respiratory illness; WHO, World Health Organization.

### Laboratory testing

All laboratory tests were performed in an international organization for standardization (ISO)15189 certified and Good Clinical Practice-accredited CDC-supported laboratories at KEMRI-CGHR in Kisumu and Nairobi, Kenya. The serum specimens were tested for anti- Spike IgG antibodies according to manufacturer’s instructions using the SCoV-2 Detect™ IgG ELISA kit (Catalogue #64824, InBios International, Inc, USA). The kit manufacturer reports of 92% and Specificty of 99% ^18, 19^. The NP/OP swabs were tested by rRT-PCR for SARS-CoV-2 and select positives sequenced for variant detection using partial and full genome sequencing at the CDC-supported laboratories in Kisumu.

### Statistical analysis

**Figure 3.**
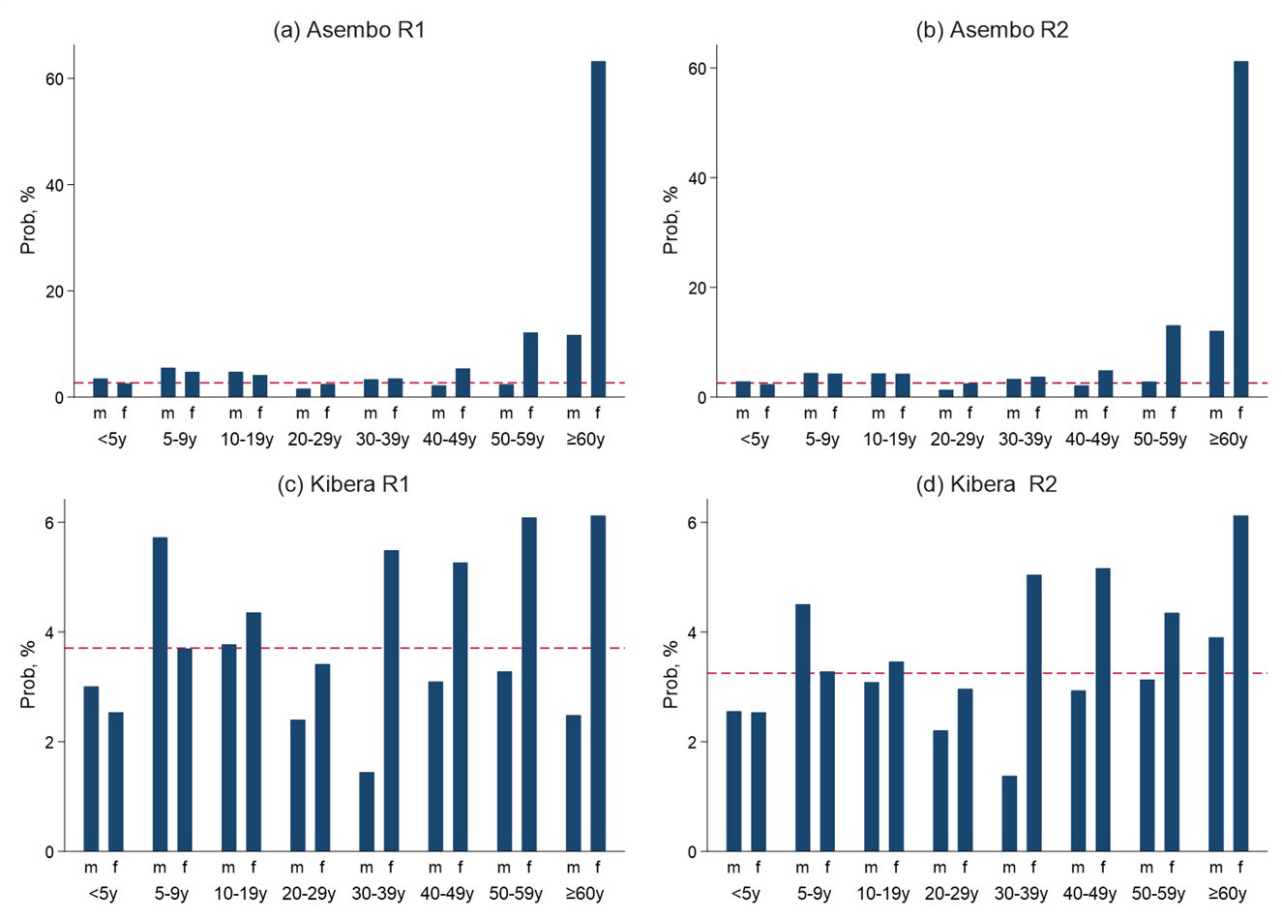
Panel figure showing the probability (prob) of an individual being selected by age and sex in Asembo and Kibera population based infectious disease surveillance (PBIDS) for seroprevalence surveys for round one and two (R1 and R2).

All data cleaning, management and analyses were performed using Stata 15.1 software (STATA Corp, Texas, USA) (free alternative, R Statistical Software) in accordance with methods we previously published ^10^. Briefly, individual seroprevalence of SARS-CoV-2 antibodies was defined as a percentage of the seropositive individuals among those tested accounting for household clustering and weighting by age and sex distribution of the PBIDS general population in each surveillance site as of March 2021 ( Figure 3). The standard errors for generating the 95% confidence intervals were computed using the Taylor linearized variance estimation method ^20^. Pearson’s chi-squared test was used to assess the association of categorical variables with individual seropositivity. Household seroprevalence (defined as the percentage of households with at least one seropositive member) was estimated and stratified by household size (usual number of persons in the household), and number of persons enrolled in the serosurvey per household. Age, sex, relationship to head of the household, main occupation, household size, and underlying medical conditions associated with serious COVID-19 complications (e.g., known hypertensive, asthmatic or diabetic) were considered in the univariable logistic regression model for determining the factors associated with individual seropositivity for each site and survey round. Age and sex were considered *a priori* for inclusion in the multivariable logistic regression. The final multivariable logistic regression model also included variables with p-value of ≤0.05 and accounted for sampling weights and clustering by household using the clustered sandwich estimator ^21, 22^. Adjusted odds ratio (aOR) and 95% confidence intervals (CI) were presented and two-sided p-values <0.05 were considered statistically significant. In addition, weekly SARS-CoV-2 positivity (percentage of RT-PCR positive cases from the total tested in a week) by rRT-PCR from the health facility surveillance is presented to provide context to the seroprevalence surveys up to 31 ^st^ December 2021.

### Ethical considerations

Individual written informed consent/verbal assent was obtained from all the study participants and/or their parents/guardian. Ethical approval for the study was provided by the KEMRI Scientific and Ethical Review Committee in Kenya (#4168, February 15 ^th^, 2021). This activity was also reviewed by CDC and was conducted consistent with applicable federal law and CDC policy as provided for in the Code of Federal Regulations (45 C.F.R part 46 and 21 C.F.R. part 56). The PBIDS protocol is approved by KEMRI Scientific and Ethical Review Committee in Kenya (#2761), Washington State University reliance agreement and CDC institutional review board (#6775).

## Results

### SARS-CoV-2 detections in the PBIDS health facilities in Asembo and Kibera

**Figure 4.**
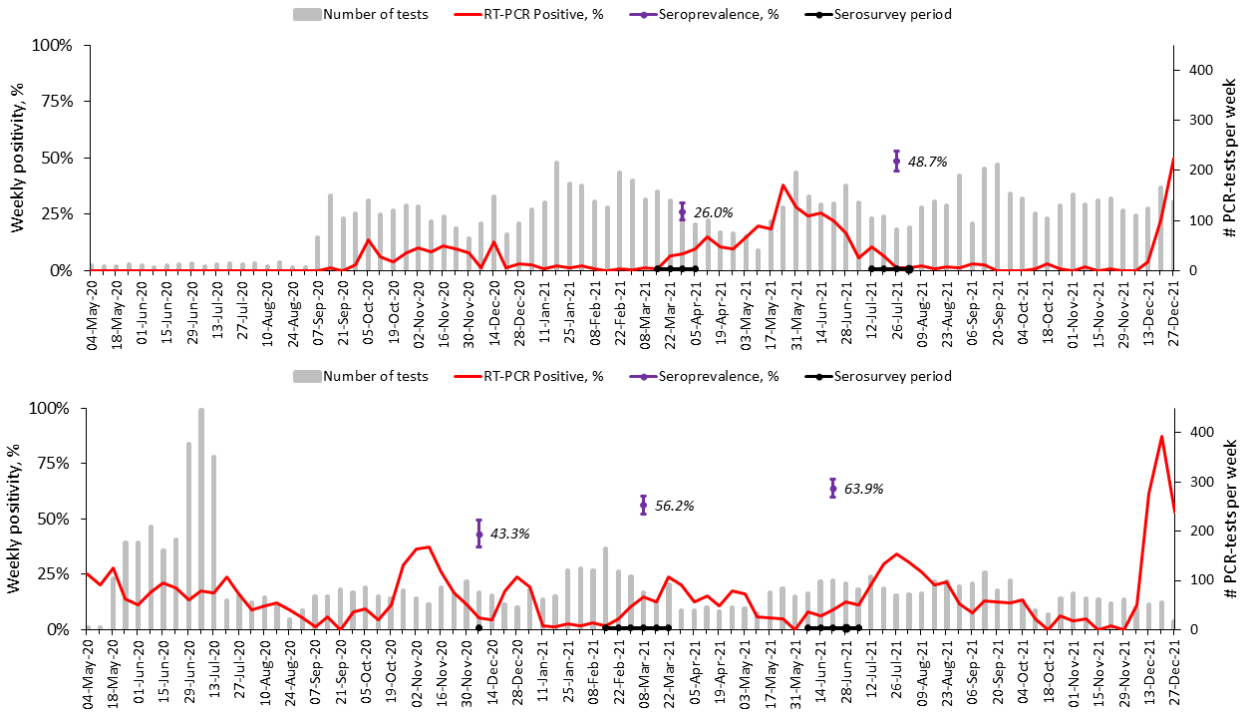
Temporal trends of weekly PCR-positivity of severe acute respiratory syndrome coronavirus 2 (SARS-CoV-2) detections in Asembo (top) and Kibera (bottom) population based infectious disease surveillance (PBIDS) from May 2020 to December 2021. Included are the overall weighted individual seropositivity in the respective times of the serosurvey in Asembo and Kibera.

From 1 ^st^ May 2020 to 31 ^st^ December 2021, SARS-CoV-2 was detected at varying intensities at different time periods, as shown in Figure 4. In Asembo, three waves were observed by 31 ^st^ December 2021: first wave in September to December 2020, second wave from March to July 2021 and the last from early December 2021 and ongoing by the end of the reporting period. In comparison, Kibera had five waves: first wave was ongoing from the beginning of the COVID-19 surveillance in May 2020 and ended in August 2020, second wave from September 2020 to January 2021, third wave from February 2021 to May 2021, fourth from June to October 2021, and fifth wave began in November 2021 and was ongoing by the end of the reporting period. The second wave in Asembo and the fourth in Kibera were predominated by the Delta variant (unpublished reports, Clayton Onyango) and started earlier in Asembo in western Kenya before being detected in Kibera in Nairobi. The latest wave was associated with Omicron (BA.1) variant (unpublished reports, Clayton Onyango) and had the highest weekly RT-PCR positivity at the peak in December 2021 of 50% in Asembo and 87% in Kibera.

### Household and participant enrolment for the seroprevalence surveys

Of the 349 randomly selected households (with 1,265 members) in Asembo, household heads in 79.1% (276 households with 1,188 eligible members) gave permission for their households to participate ( Figure 2). Of the 469 randomly selected households (with 1,589 members), household heads in 64.6% (294 households with 1,415 eligible members) agreed to participate in Kibera. Of the 76 households in Asembo that did not participate, 37 (48.7%) declined participation, 13 (17.1%) did not have a household head available for consenting, 11 (14.5%) had moved, and 12 (15.8%) provided no reason for not participating. In Kibera, 172 households did not accept to participate; 44 (25.6%) declined participation, 56 (32.6%) did not have a household head available for consenting, 63 (36.6%) had moved, and 12 (0.6%) provided no reason. Individual written informed consent was obtained from 955 (67.5% of eligible members) and 882 (74.2%) participants in Asembo and Kibera, respectively. Of the 233 who didn’t consent in Asembo, 115 (49.4%) had out-migrated, 69 (29.6%) were not found at home, even after three attempts, 47 (20.2%) declined and two (0.9%) provided no reason. In Kibera, 533 did not consent; 347 (65.1%) were not found at home, 145 (27.2%) declined participation, 6 (1.1%) had out-migrated and 35 (6.6%) provided no reason.

The median number of individuals in a household was three (interquartile range (IQR), 1–5; range, 1–15) in Asembo, and three (IQR, 1–5; range, 1–19) in Kibera while the corresponding median number of consented individuals per household was three (IQR, 2–5; range, 1–13) in Asembo and three (IQR, 1–4; range, 1–11) in Kibera.

In Asembo, female participants aged 50 years and above, male participants over 60 years and both sexes aged 5–19 years old were overrepresented, while men aged 20–29 years were underrepresented in the two surveys, relative to the PBIDS population ( Figure 3). For Kibera, male children aged 5–9 years and women aged 30 years and above were overrepresented, while both female and male children below 5 years of age and men aged 30–59 years were underrepresented in both surveys, compared to the PBIDS general population ( Figure 3).

### Timing and participant characteristics in the seroprevalence surveys

The first serosurvey (round one, R1) was conducted between 19 ^th^ February and 28 ^th^ March 2021 (median date, 11 ^th^ March 2021), and the second round (R2) between 4 ^th^ June to 4 ^th^ July 2021 (median date, 15 ^th^ June 2021) yielding 859 and 750 serum specimens, respectively, in Kibera ( Table 2). In Asembo, 935 and 888 sera specimens were collected in the first (12 ^th^ March to 8 ^th^ April 2021, median date, 25 ^th^ March 2021) and second (8 ^th^ July to 4 ^th^ August 2021, median date 19 ^th^ July 2021) serosurvey, respectively. The median number of sampled individuals per household was three (IQR, 2–5; range, 1–11) in Asembo and three (IQR, 1–4; range, 1–11) in Kibera. During the second round of serosurveys, consent was obtained for an additional 115 and 160 individuals and they were sampled from the participating households in Asembo and Kibera, respectively. These were mainly individuals who were not available in the households for enrolment during the first round of the serosurvey.

**Table 2.** Participant and household characteristics for the March and June 2021 serosurveys in Asembo, Siaya County and Kibera, Nairobi County, Kenya.

Characteristics	Categories	Asembo				Kibera			
		*Round 1,* * R1*		*Round 2,* * R2*		*Round 1,* * R1*		*Round 2,* * R2*	
		n	*%*	*n*	*%*	*n*	*%*	*n*	*%*
A. Individuals									
Overall	All	935	100	888	100	859	100	750	100
Age group, years	0–4	79	8.4	68	7.7	73	8.5	67	8.9
	5–9	145	15.5	122	13.7	133	15.5	110	14.7
	10–19	273	29.2	261	29.4	250	29.1	201	26.8
	20–29	97	10.4	93	10.5	140	16.3	124	16.5
	30–39	110	11.8	114	12.8	119	13.9	110	14.7
	40–49	78	8.3	72	8.1	88	10.2	85	11.3
	50–59	58	6.2	64	7.2	43	5.0	36	4.8
	60+	95	10.2	94	10.6	13	1.5	17	2.3
Sex	Male	417	44.6	377	42.5	366	42.6	319	42.5
	Female	518	55.4	511	57.5	493	57.4	431	57.5
Relationships	Household head	219	23.4	210	23.6	169	19.7	122	16.3
	Spouse	129	13.8	124	14.0	157	18.3	162	21.6
	Children	457	48.9	439	49.4	499	58.1	434	57.9
	Grandchildren	97	10.4	93	10.5	15	1.7	18	2.4
	Other relatives	33	3.5	22	2.5	19	2.2	14	1.9
Occupation	Unemployed	581	62.1	421	47.4	432	50.3	342	45.6
	In school	56	6.0	56	6.3	151	17.6	94	12.5
	Employed - informal	27	2.9	21	2.4	6	0.7	0	0.0
	Employed - formal	51	5.5	145	16.3	119	13.9	142	18.9
	Self employed	220	23.5	190	21.4	151	17.6	115	15.3
	Missing data	0	0.0	55	6.2	0	0.0	57	7.6
Underlying medical condition	No	816	87.3	844	95.0	806	93.8	713	95.1
	Yes	119	12.7	44	5.0	53	6.2	37	4.9
	Asthma	17	1.8	14	1.6	12	1.4	3	0.4
	Hypertension	34	3.6	16	1.8	27	3.1	25	3.3
	Diabetes	9	1.0	5	0.6	2	0.2	3	0.4
	Other	66	7.1	13	1.5	14	1.6	7	0.9
Education Level	No formal education	187	20.0	128	14.4	132	15.4	85	11.3
	Primary	518	55.4	535	60.2	428	49.8	418	55.7
	Secondary	196	21.0	190	21.4	230	26.8	175	23.3
	Post-secondary	34	3.6	35	3.9	69	8.0	72	9.6
B. Households									
	All	276	100.0	262	100.0	294	100.0	252	100.0
Household size: Number of individuals per household	1	35	12.7	41	15.6	26	8.8	38	15.1
	2	42	15.2	44	16.8	21	7.1	30	11.9
	3	44	15.9	43	16.4	36	12.2	54	21.4
	4	40	14.5	37	14.1	57	19.4	61	24.2
	5	40	14.5	45	17.2	49	16.7	33	13.1
	≥6	75	27.2	52	19.8	105	35.7	36	14.3
Household size	Median (IQR)	4 (2-6)		4 (2-5)		5 (3-6)		4(2-5)	
Number of individuals sampled per household	1	53	19.2	52	19.8	84	28.6	61	24.2
	2	55	19.9	48	18.3	51	17.3	48	19.0
	3	50	18.1	48	18.3	58	19.7	50	19.8
	4	45	16.3	43	16.4	46	15.6	51	20.2
	5	32	11.6	33	12.6	34	11.6	24	9.5
	≥6	41	14.9	38	14.5	21	7.1	18	7.1
Number of individuals sampled per household	Median (IQR)		3 (2-5)		3 (2-5)		3 (1-4)		3 (2-4)	

The majority of the sampled individuals during the two rounds in both sites were female (55.4% in Asembo and 57.5% in Kibera in the first round, Table 2). The average age in years for participants was higher for Asembo participants compared to Kibera participants (26.6 (95% CI: 25.3, 28.0) *versus* 22.7 (95% CI: 21.7, 23.8) years during the first serosurvey).

### Individual seroprevalence of SARS-CoV-2 antibodies

Of the tested individuals, 234 (25.0%) and 440 (49.5%) were seropositive in Asembo for the first and second serosurvey, respectively. Correspondingly, 478 (55.6%) and 483 (64.4%) individuals were seropositive in the respective rounds in Kibera. The overall weighted individual seroprevalence increased from 56.2% (95% CI: 52.1, 60.2%) in March 2021 to 63.9% (95% CI: 59.5, 68.0%) in June 2021 in Kibera. For Asembo, the weighted seroprevalence almost doubled from 26.0% (95% CI: 22.4, 30.0%) in March 2021 to 48.7% (95% CI: 44.3, 53.2%) in July 2021. At the respective time points for the serosurvey, Kibera had consistently higher seroprevalence than Asembo. In both sites and for each of the two rounds, the seroprevalence did not vary by sex, but was lower in children compared to adults ( Table 3). An appreciable increase in age-specific individual seroprevalence over the rounds was observed in all age groups in Asembo (14.3–28.5% increase) and among young participants in the 5–19 years age groups (11.8–17.7%) in Kibera.

**Table 3.** Crude and weighted individual seroprevalence in Asembo, Siaya County and Kibera, Nairobi County, Kenya.

Characteristics	Categories	Round	Asembo								Kibera							
			*N*	*n*	*Crude*			*Weighted*			*N*	*n*	*Crude*			*Weighted*		
					*P, %*	*95% CI*		*P, %*	*95% CI*				*P, %*	*95% CI*		*P, %*	*95% CI*	
Overall	**All**	1	935	234	25.0	22.3	27.9	26.0	22.4	30.0	859	478	55.6	52.3	59.0	56.2	52.1	60.2
		2	888	440	49.5	46.2	52.9	48.7	44.3	53.2	750	483	64.4	60.9	67.8	63.9	59.6	67.9
Age group, years	0–4	1	79	14	17.7	10.0	27.9	17.8	10.4	28.6	73	23	31.5	21.1	43.4	31.6	21.7	43.6
		2	68	22	32.4	21.5	44.8	32.1	22.3	43.8	67	25	37.3	25.8	50.0	37.3	26.5	49.5
	5–9	1	145	20	13.8	8.6	20.5	14.0	9.4	20.3	133	45	33.8	25.9	42.5	35.2	26.6	44.9
		2	122	47	38.5	29.9	47.8	38.4	30.2	47.4	110	57	51.8	42.1	61.4	52.9	43.1	62.4
	10–19	1	273	80	29.3	24.0	35.1	29.3	23.7	35.6	250	148	59.2	52.8	65.4	59.2	52.4	65.7
		2	261	151	57.9	51.6	63.9	57.9	51.2	64.2	201	143	71.1	64.4	77.3	71.1	64.2	77.1
	20–29	1	97	27	27.8	19.2	37.9	27.9	19.7	37.9	140	90	64.3	55.8	72.2	64.9	56.2	72.7
		2	93	46	49.5	38.9	60.0	48.5	38.0	59.0	124	88	71.0	62.1	78.8	71.7	62.6	79.4
	30–39	1	110	40	36.4	27.4	46.1	36.3	27.3	46.4	119	79	66.4	57.2	74.8	66.5	55.7	75.7
		2	114	64	56.1	46.5	65.4	56.1	46.8	65.1	110	73	66.4	56.7	75.1	64.3	52.5	74.5
	40–49	1	78	20	25.6	16.4	36.8	25.7	16.4	38.0	88	57	64.8	53.9	74.7	65.0	54.4	74.3
		2	72	34	47.2	35.3	59.3	49.3	37.4	61.3	85	62	72.9	62.2	82.0	72.8	62.4	81.2
	50–59	1	58	15	25.9	15.3	39.0	22.1	11.4	38.4	43	28	65.1	49.1	79.0	61.9	46.3	75.4
		2	64	31	48.4	35.8	61.3	42.4	28.2	58.1	36	23	63.9	46.2	79.2	60.9	43.6	75.8
	60+	1	95	18	18.9	11.6	28.3	20.3	11.5	33.4	13	8	61.5	31.6	86.1	72.2	43.7	89.7
		2	94	45	47.9	37.5	58.4	47.4	34.6	60.5	17	12	70.6	44.0	89.7	71.2	45.9	87.8
Sex	Male	1	417	100	24.0	20.0	28.4	25.0	20.2	30.5	366	191	52.2	46.9	57.4	56.0	50.1	61.8
		2	377	188	49.9	44.7	55.0	48.8	42.8	54.9	319	194	60.8	55.2	66.2	62.0	55.8	67.7
	Female	1	518	134	25.9	22.1	29.9	27.0	22.4	32.1	493	287	58.2	53.7	62.6	56.4	51.5	61.1
		2	511	252	49.3	44.9	53.7	48.6	43.0	54.3	431	289	67.1	62.4	71.5	65.7	60.8	70.4
Highest education level	None	1	187	27	14.4	9.7	20.3	15.3	10.5	21.7	132	39	29.5	21.9	38.1	30.4	22.1	40.3
		2	128	44	34.4	26.2	43.3	32.4	24.0	42.1	85	34	40.0	29.5	51.2	38.7	28.0	50.5
	Primary	1	518	135	26.1	22.3	30.1	26.2	21.6	31.5	428	238	55.6	50.8	60.4	56.4	51.0	61.7
		2	535	271	50.7	46.3	55.0	50.3	44.7	55.9	418	273	65.3	60.5	69.9	64.8	59.7	69.6
	Secondary	1	196	59	30.1	23.8	37.0	30.5	23.6	38.4	230	154	67.0	60.5	73.0	66.6	59.6	72.8
		2	190	103	54.2	46.8	61.4	51.2	43.5	58.8	175	129	73.7	66.5	80.1	72.8	64.4	79.8
	Post-secondary	1	34	13	38.2	22.2	56.4	41.7	25.4	60.1	69	47	68.1	55.8	78.8	68.5	55.4	79.2
		2	35	22	62.9	44.9	78.5	66.3	48.2	80.7	72	47	65.3	53.1	76.1	68.3	55.0	79.1
Relationship to the household (HH) head	Head	1	219	62	28.3	22.4	34.8	29.9	22.7	38.1	169	100	59.2	51.4	66.7	59.6	51.1	67.7
		2	210	98	46.7	39.8	53.7	45.2	36.6	54.0	122	81	66.4	57.3	74.7	62.3	52.1	71.6
	Spouse	1	129	34	26.4	19.0	34.8	31.5	22.7	41.9	157	111	70.7	62.9	77.7	70.9	62.9	77.7
		2	124	72	58.1	48.9	66.9	61.1	50.6	70.7	162	112	69.1	61.4	76.1	70.0	62.0	76.9
	Children	1	457	110	24.1	20.2	28.3	24.5	19.8	29.8	499	253	50.7	46.2	55.2	52.1	47.1	57.1
		2	439	217	49.4	44.7	54.2	47.7	41.9	53.6	434	271	62.4	57.7	67.0	62.9	57.6	67.9
	Grand children	1	97	23	23.7	15.7	33.4	23.7	16.9	32.1	15	4	26.7	7.8	55.1	25.6	10.1	51.2
		2	93	45	48.4	37.9	59.0	50.0	38.4	61.6	18	10	55.6	30.8	78.5	57.9	35.8	77.2
	Others	1	33	5	15.2	5.1	31.9	17.2	7.1	36.2	19	10	52.6	28.9	75.6	54.1	30.1	76.4
		2	22	8	36.4	17.2	59.3	34.8	15.9	60.1	14	9	64.3	35.1	87.2	65.7	37.5	86.0
Underlying medical condition	No	1	816	209	25.6	22.6	28.8	26.3	22.4	30.7	806	448	55.6	52.1	59.0	56.2	52.0	60.3
		2	844	419	49.6	46.2	53.1	48.7	44.2	53.2	713	466	65.4	61.7	68.9	64.8	60.5	68.9
	Yes	1	119	25	21.0	14.1	29.4	23.2	15.9	32.6	53	30	56.6	42.3	70.2	56.1	40.3	70.7
		2	44	21	47.7	32.5	63.3	50.2	29.3	71.0	37	17	45.9	29.5	63.1	41.2	25.9	58.5
Household size	1	1	35	7	20.0	8.4	36.9	12.6	4.5	30.8	26	18	69.2	48.2	85.7	71.4	50.7	85.8
		2	41	24	58.5	42.1	73.7	56.6	36.0	75.1	38	25	65.8	48.6	80.4	58.6	40.2	74.9
	2	1	75	21	28.0	18.2	39.6	33.8	22.0	47.9	30	17	56.7	37.4	74.5	63.4	44.5	78.9
		2	83	38	45.8	34.8	57.1	53.8	41.3	65.9	50	35	70.0	55.4	82.1	73.1	57.6	84.4
	3	1	108	33	30.6	22.1	40.2	34.3	23.5	47.0	77	38	49.4	37.8	61.0	51.1	36.7	65.3
		2	119	65	54.6	45.2	63.8	42.7	30.8	55.5	127	72	56.7	47.6	65.5	56.4	45.9	66.3
	4	1	132	26	19.7	13.3	27.5	18.8	11.6	28.9	153	91	59.5	51.3	67.3	61.1	50.9	70.5
		2	132	71	53.8	44.9	62.5	55.6	44.4	66.3	204	126	61.8	54.7	68.5	60.8	52.8	68.3
	5	1	165	50	30.3	23.4	37.9	31.5	24.0	40.1	163	85	52.1	44.2	60.0	50.4	41.5	59.3
		2	192	108	56.3	48.9	63.4	54.2	45.9	62.2	140	86	61.4	52.8	69.5	60.6	50.5	69.8
	≥6	1	420	97	23.1	19.1	27.4	24.3	18.9	30.7	410	229	55.9	50.9	60.7	56.0	50.2	61.7
		2	321	134	41.7	36.3	47.4	43.3	35.9	51.0	191	139	72.8	65.9	79.0	73.4	65.4	80.0
Number of samples per HH	1	1	53	12	22.6	12.3	36.2	21.6	9.9	40.9	84	55	65.5	54.3	75.5	68.9	57.5	78.3
		2	52	32	61.5	47.0	74.7	61.9	43.2	77.7	61	46	75.4	62.7	85.5	70.2	55.1	81.9
	2	1	110	26	23.6	16.1	32.7	25.3	16.9	36.0	102	56	54.9	44.7	64.8	56.3	44.4	67.6
		2	96	43	44.8	34.6	55.3	48.1	37.1	59.3	96	58	60.4	49.9	70.3	61.2	49.2	72.0
	3	1	150	43	28.7	21.6	36.6	30.1	22.1	39.6	173	98	56.6	48.9	64.1	56.9	47.7	65.6
		2	144	74	51.4	42.9	59.8	43.3	32.6	54.5	150	87	58.0	49.7	66.0	58.1	49.4	66.4
	4	1	180	42	23.3	17.4	30.2	24.3	16.3	34.7	183	91	49.7	42.3	57.2	48.9	40.4	57.5
		2	172	93	54.1	46.3	61.7	54.9	45.5	64.0	203	127	62.6	55.5	69.2	61.9	53.5	69.5
	5	1	160	43	26.9	20.2	34.4	26.5	19.0	35.6	170	87	51.2	43.4	58.9	50.6	42.4	58.7
		2	165	87	52.7	44.8	60.5	50.6	41.0	60.2	120	77	64.2	54.9	72.7	62.2	52.4	71.2
	≥6	1	282	68	24.1	19.2	29.5	25.7	19.1	33.7	147	91	61.9	53.5	69.8	62.5	51.3	72.5
		2	259	111	42.9	36.7	49.1	44.6	36.0	53.5	120	88	73.3	64.5	81.0	74.7	62.7	83.8
Household sizes, Grouped	1-2	1	77	26	33.8	23.4	45.4	26.4	17.4	37.8	47	32	68.1	52.9	80.9	67.2	53.4	78.5
		2	85	52	61.2	50.0	71.6	54.7	43.8	65.2	68	51	75.0	63.0	84.7	66.4	54.2	76.7
	3-4	1	84	40	47.6	36.6	58.8	25.0	18.4	33.1	93	73	78.5	68.8	86.3	57.4	49.0	65.5
		2	80	66	82.5	72.4	90.1	49.8	41.2	58.3	115	102	88.7	81.4	93.8	59.1	52.7	65.2
	≥5	1	115	79	68.7	59.4	77.0	26.4	21.8	31.5	154	132	85.7	79.2	90.8	54.4	49.5	59.2
		2	97	90	92.8	85.7	97.0	47.2	41.5	52.9	69	67	97.1	89.9	99.6	68.0	61.6	73.9
Main occupation	None	1	581	130	22.4	19.0	26.0	23.0	19.0	27.6	432	218	50.5	45.6	55.3	50.9	45.7	56.1
		2	421	196	46.6	41.7	51.4	44.8	39.5	50.2	342	210	61.4	56.0	66.6	60.8	55.0	66.4
	School going	1	56	19	33.9	21.8	47.8	33.3	22.0	46.9	151	84	55.6	47.3	63.7	57.7	47.7	67.2
		2	56	36	64.3	50.4	76.6	62.8	45.7	77.1	94	62	66.0	55.5	75.4	66.8	56.6	75.7
	Informal employ	1	27	7	25.9	11.1	46.3	32.7	14.7	57.8	6	5	83.3	35.9	99.6	90.3	49.9	98.9
		2	21	11	52.4	29.8	74.3	41.1	16.0	72.0	0							
	Formal employ	1	51	21	41.2	27.6	55.8	39.0	24.4	55.8	119	69	58.0	48.6	67.0	66.0	56.6	74.3
		2	145	87	60.0	51.5	68.0	61.5	52.2	70.0	142	92	64.8	56.3	72.6	56.6	46.7	66.1
	Self-employed	1	220	57	25.9	20.3	32.2	27.1	20.3	35.2	151	102	67.5	59.5	74.9	73.6	63.3	81.8
		2	190	91	47.9	40.6	55.2	48.1	38.8	57.5	115	87	75.7	66.8	83.2	67.8	59.1	75.5
	Unknown	1																
		2	55	19	34.5	22.2	48.6	31.1	20.2	44.6	57	32	56.1	42.4	69.3	55.4	42.2	67.8

N, total number; n, Number seropositive; Crude, Crude individual seroprevalence; weighted, Weighted individual seroprevalence accounting for sampling weights and household clustering; CI, Confidence Interval.

In Kibera, the age effect was also observed for seroprevalence estimates by relationship to the household head in the first round with grandchildren (25.0%; 95% CI: 10.1, 51.2%) and children (52.1%; 95% CI: 47.1, 57.1%) registering lower estimates compared to the household head (59.6%; 95% CI: 51.1, 67.7 %) and other adults including spouses (70.9%; 95% CI: 62.9, 77.7%) and other relatives (54.1%; 95% CI: 30.1, 76.4%). However, these differences in seroprevalence by relationships were not statistically significant in the second serosurvey in Kibera as well as in both rounds in Asembo.

Seroprevalence in each round and site increased with the level of education, with the lowest among those with no education and highest among those with secondary or above level of education—again highlighting the age effect as the young children were coded to have no education. There was no obvious pattern in seroprevalence based on main occupation groups ( Table 3) for the two sites and over the two survey rounds. Participants with any underlying medical conditions had similar seroprevalence as those without in each round and site ( Table 3).

COVID-19 vaccine uptake was low at the time these serosurveys were conducted. None of the study participants had received the COVID-19 vaccine before round one of the serosurvey in both sites. By round two, 11 (1.2%) participants reported having received the COVID-19 vaccine in Asembo, while in Kibera, 12 (1.6%) reported receipt of the vaccine—only one participant in Asembo and three participants in Kibera reported receipt of two doses. All the vaccinated participants had received Oxford-AstraZeneca vaccine except for one participant from Kibera who reported receiving Johnson & Johnson COVID-19 vaccine. Of the 23 individuals who reported having received the COVID-19 vaccine in round two of the serosurvey, 18 (78.3%) were seropositive—nine from each site. Among those reporting receipt of COVID-19 vaccine, seropositivity was 81.8% in Asembo and 75.0% in Kibera. The median duration (IQR) for those seronegative after receiving COVID-19 vaccine in both sites (n=5) was 42 (4-72) days compared to 63.5 (3-100) days for those seropositive, though not statistically significant - Wilcoxon rank-sum test p-value=0.5490. 

### Household seroprevalence of SARS-CoV-2 antibodies

Samples tested were from 276 and 262 households in Asembo and 294 and 252 in Kibera for round one and two, respectively ( Table 4). Among sampled households in Asembo, 145 (52.5%; 95% CI: 46.5–58.6%) and 208 (79.4%; 95% CI: 74.0–84.1%) had at least one seropositive participant in the first and second surveys, respectively. In Kibera, 237 households (80.6%; 95% CI: 75.6–85.0%) and 220 households (87.3%; 95% CI: 82.5–91.1%) had at least one seropositive participant in round one and two, respectively. Within households with at least one seropositive individual, the median (IQR) percentage of seropositive individuals in the household was 40.0% (25.0–60.0%) and 63.3% (40.0–100%) in Asembo for round one and two, respectively. The corresponding median (IQR) estimates for Kibera were higher at 75.0% (50.0–100%) and 80.0% (50.0–100%). Of 223 and 210 households with two or more members enrolled in Asembo, 133 (59.6%) and 176 (83.8%) had at least one person seropositive in the respective survey rounds. On the other hand, the mean seropositivity among households with at least one seropositive person and two or more participants enrolled in Asembo was 42.3% (95% CI: 38.4–46.1%) and 58.6% (95% CI: 54.7–62.6%) in round one and two of the serosurvey, respectively. For Kibera, the corresponding mean seropositivity in the similar households was 62.7% (95% CI: 58.9–66.6%) and 68.1% (95% CI: 64.3–71.9%) in round one and two of the serosurvey, respectively.

**Table 4.** Household characteristics and prevalence of households with seropositive individuals in Asembo, Siaya County and Kibera, Nairobi County, Kenya.

Characteristics	Categories	Round	Asembo					Kibera				
			*N ^1^*	*n ^2^*	*HH P ^3^, %*	*95% CI*		*N*	*n*	*HH P, %*	*95% CI*	
Overall	All	1	276	145	52.5	46.5	58.6	294	237	80.6	75.6	85.0
		2	262	208	79.4	74.0	84.1	252	220	87.3	82.5	91.1
Household size	1	1	35	7	20.0	8.4	36.9	26	18	69.2	48.2	85.7
		2	41	24	58.5	42.1	73.7	38	25	65.8	48.6	80.4
	2	1	42	19	45.2	29.8	61.3	21	14	66.7	43.0	85.4
		2	44	28	63.6	47.8	77.6	30	26	86.7	69.3	96.2
	3	1	44	22	50.0	34.6	65.4	36	25	69.4	51.9	83.7
		2	43	35	81.4	66.6	91.6	54	44	81.5	68.6	90.7
	4	1	40	18	45.0	29.3	61.5	57	48	84.2	72.1	92.5
		2	37	31	83.8	68.0	93.8	61	58	95.1	86.3	99.0
	5	1	40	31	77.5	61.5	89.2	49	43	87.8	75.2	95.4
		2	45	43	95.6	84.9	99.5	33	31	93.9	79.8	99.3
	6	1	75	48	64.0	52.1	74.8	105	89	84.8	76.4	91.0
		2	52	47	90.4	79.0	96.8	36	36	100.0	90.3	100.0
Household size, grouped	1-2	1	77	26	33.8	23.4	45.4	47	32	68.1	52.9	80.9
		2	85	52	61.2	50.0	71.6	68	51	75.0	63.0	84.7
	3-4	1	84	40	47.6	36.6	58.8	93	73	78.5	68.8	86.3
		2	80	66	82.5	72.4	90.1	115	102	88.7	81.4	93.8
	≥5	1	115	79	68.7	59.4	77.0	154	132	85.7	79.2	90.8
		2	97	90	92.8	85.7	97.0	69	67	97.1	89.9	99.6

1, Number of households (N); 2, number of households with a seropositive individual (n); 3, percentage of households with a seropositive individual *i.e.*, household seroprevalence (HH P%), 95% CI, Confidence interval; HH, household.

### Risk factors for individual seropositivity

Age was a predictor of individual seropositivity. Relative to adults aged 20–29 years, young age groups (<20 years) had lower odds of being seropositive in the respective sites and rounds. The odds of being seropositive were similar for older adults (age groups ≥30 years) compared to the reference group, 20–29 years. The odds of being seropositive among the elderly groups (≥60 years) were not different from the referent 20–29 years age group across the sites and by survey round. Relative to household heads, spouses had higher odds of being seropositive in Asembo round two (adjusted Odds Ratio, aOR, 2.47; 95% CI: 1.35–4.54) and Kibera round one (aOR; 1.89 (95% CI: 1.08–3.31). During round two of serosurvey in Asembo, the odds of seropositivity were higher in the employed participants compared to those not employed, aOR, 3.37 (95% CI: 1.76–6.45). This association of employment status and seropositivity was not observed in Kibera. Sex and highest level of education were not significantly associated with individual seropositivity for any of the sites or rounds ( Table 5).

**Table 5.** Risk factors for individual seropositivity from multivariable logistic regression model in Asembo, Siaya County and Kibera, Nairobi County, Kenya.

Risk factors	Categories	Asembo								Kibera							
		*Round 1*				*Round 2*				*Round 1*				*Round 2*			
		*OR*	*95% CI*		*P-value*	*OR*	*95% CI*		*P-value*	*OR*	*95% CI*		*P-value*	*OR*	*95% CI*		*P-value*
Age group in years	<5	0.59	0.25	1.41	0.233	0.94	0.44	2.00	0.878	**0.23**	**0.11**	**0.46**	**<0.001**	**0.21**	**0.10**	**0.46**	**<0.001**
	5-9	**0.42**	**0.20**	**0.90**	**0.027**	1.15	0.57	2.33	0.692	**0.25**	**0.14**	**0.46**	**<0.001**	**0.38**	**0.18**	**0.79**	**0.01**
	10-19	1.06	0.56	2.01	0.861	**2.41**	**1.28**	**4.52**	**0.006**	0.66	0.38	1.15	0.143	0.83	0.40	1.69	0.603
	20-29	1.00				1.00				1.00				1.00			
	30-39	1.28	0.63	2.60	0.488	1.01	0.50	2.04	0.982	1.51	0.81	2.84	0.195	0.89	0.46	1.73	0.737
	40-49	0.84	0.35	1.98	0.688	0.81	0.39	1.69	0.58	1.57	0.78	3.16	0.208	1.57	0.75	3.29	0.23
	50-59	0.66	0.24	1.86	0.432	0.68	0.27	1.72	0.409	1.36	0.57	3.24	0.488	0.93	0.37	2.35	0.874
	≥60	0.61	0.24	1.57	0.303	1.01	0.42	2.42	0.978	1.93	0.50	7.41	0.334	2.10	0.56	7.87	0.271
Sex	Male	1.00				1.00				1.00				1.00			
	Female	1.06	0.74	1.52	0.755	0.94	0.67	1.31	0.704	0.91	0.67	1.24	0.559	1.12	0.81	1.56	0.493
Relationship to household head	HH head	1.00				1.00				1.00				1.00			
	Spouse	1.02	0.55	1.87	0.955	**2.47**	**1.35**	**4.54**	**0.004**	**1.89**	**1.08**	**3.31**	**0.026**	1.47	0.75	2.88	0.259
	Children	0.82	0.39	1.71	0.592	1.51	0.72	3.13	0.272	1.80	0.90	3.62	0.098	1.83	0.86	3.88	0.115
	Grandchild	0.88	0.36	2.15	0.785	1.82	0.74	4.46	0.191	1.05	0.26	4.24	0.949	2.12	0.57	7.87	0.258
	Others	0.49	0.15	1.67	0.255	0.87	0.24	3.11	0.831	1.24	0.39	3.94	0.721	1.41	0.40	5.00	0.593
Existing condition	No	1.00				1.00				1.00				1.00			
	Yes	0.79	0.45	1.39	0.417	0.80	0.30	2.15	0.661	0.82	0.42	1.63	0.573	**0.27**	**0.12**	**0.58**	**0.001**
Household size, grouped	1-2	1.00	0.51	1.96	0.99	1.27	0.70	2.29	0.427	1.24	0.62	2.48	0.55	1.17	0.64	2.15	0.608
	3-4	1.00				1.00				1.00				1.00			
	≥5	1.15	0.71	1.86	0.572	0.93	0.61	1.42	0.736	0.83	0.55	1.27	0.388	1.38	0.92	2.08	0.123
Main occupation	None	1.00				1.00				1.00				1.00			
	In school	1.61	0.89	2.91	0.113	1.81	0.88	3.72	0.107	1.23	0.77	1.97	0.39	1.11	0.64	1.90	0.716
	Employed	1.52	0.71	3.23	0.281	**3.37**	**1.76**	**6.45**	**<0.001**	0.66	0.37	1.15	0.14	1.02	0.55	1.91	0.939
	Self emp	0.94	0.50	1.76	0.851	1.87	0.96	3.66	0.065	0.99	0.55	1.77	0.963	1.48	0.73	3.02	0.275
	Missing	-	-	-	-	0.58	0.30	1.12	0.104					1.07	0.59	1.96	0.815

95% CI, Confidence interval; HH, household.

### Duration of seropositivity

Of the individuals enrolled in round one, 754 (80.6%) in Asembo and 606 (70.6%) in Kibera participated in the second serosurvey. The median duration between the serosurveys was longer in Asembo (3.9 months; IQR, 3.6–4.0 months) than in Kibera (3.2 months; IQR, 2.9–3.4) months). Between the two serosurveys, 215/564 (38.1%) of the previously seronegative participants seroconverted in Asembo (median age, 18.1 (IQR, 11.8–40.1 years) and 98/273 (35.9%) in Kibera (median age, 12.1 (IQR, 6.7–25.1 years). Conversely, 29/190 (15.3%) of the previously seropositive participants returned a negative result in the second serosurvey in Asembo (median age, 23.2 (IQR, 12.5–38.3 years), whereas in Kibera 40/333 (12.0%) sero-reverted (median age, 32.8 (IQR, 23.7–40.6 years). There was no change in serostatus in 161/190 (84.7%) with median age of 21.7 (IQR, 14.2–42.9 years), and 293/333 (88.0%) with median age of 22.3 (IQR, 12.4–36.3 years), of participants who were previously seropositive, and 349/564 (61.9%) with median age, 19.1 (IQR, 9.8–45.4 years), and 175/273 (64.1%) with median age, 12.1 (IQR, 6.7–25.1 years), of those who were previously seronegative, in Asembo and Kibera, respectively. There were statistically significant differences in median ages by change or no change in the serostatus in Asembo. In Kibera, there was age dependent pattern with median age increasing from lowest in those not seroconverting, followed by those seroconverting and those non sero-reverting, to highest in those sero-reverting.

## Discussion

Leveraging on well-characterized populations in two diverse geographical locations, we report heterogenous population exposure based on settings and characteristics at 12- and 16-months following SARS-CoV-2 detection in Kenya. By the third quarter of 2021, about two-thirds of the population in an urban informal settlement in Nairobi, the capital city of Kenya, and close to half of the population in rural Asembo in western Kenya had serologic evidence of SARS-CoV-2 infection. Varying patterns of introduction and transmission of the virus in the two sites could potentially explain the observed differences in the overall seroprevalence—three waves were observed in the sparsely populated rural settings in western Kenya compared to five waves in the densely populated urban informal settlement in Nairobi by 31 ^st^ December 2021. The timing of virus introduction and transmission varied across the two sites. For instance, the first COVID-19 case was not detected until September 2020 in the rural site and the Delta wave seemed to begin in western Kenya before spreading to the urban informal settlement in Nairobi. The five waves observed in Kibera mirrored the national data ^23^ with all the five variants of concern ( *Alpha*, *Beta*, *Delta*, *Gamma and Omicron*) being detected (unpublished reports, Clayton Onyango). Vaccination coverage in both sites was very low at the time of the serosurveys with only a few (1.2–1.6%) of the study participants having received a COVID-19 vaccine by the start of the third quarter of 2021.

Recent pooled estimates from a meta-analysis of population-based studies show a seroprevalence of 65% in Africa by the third quarter of 2021 ^24^ and 45% globally ^25^, which is comparable to our estimates in the two populations in Kenya. Considerable increase in seroprevalence was documented after the emergence of new variants, mainly the Beta and Delta variants, in the African and global studies ^24– 26^. Following the introduction and spread of the Delta Variant, known to be more transmissible than the earlier variants, we observed a doubling of seroprevalence in Asembo. With vaccination coverage being low during the study period (1.2% in Asembo and 1.6% in Kibera), our seroprevalence estimates were mainly driven by infections. The urban site was yet to fully experience the Delta variant (unlike the rural site) and another serosurvey after the Delta and more recent Omicron variant circulation would likely show considerable increase in seroprevalence across the sites. Other seroprevalence studies in Kenya have documented the remarkable increase in population exposure to SARS-CoV-2 over time ^10, 15, 16, 27– 31^. Investigators at Kenya Medical Research Institute in Kilifi reported SARS-CoV-2 antibody seroprevalence among Kenyan blood donors was 49% in March 2021 and 73% in August 2021 ^16^. Considerable levels of serologic evidence of infection have also been reported for health care workers (43.8% in Nairobi) ^28^, truck drivers (43.3%) ^29^ and pregnant women seeking antenatal care services (49.9% from Kenyatta National Hospital in Nairobi) ^30^ by the end of 2020. Additional seroprevalence surveys in multiple populations after the Omicron wave would be needed to generate a more generalizable estimate of the seroprevalence across the country.

The distribution of SARS-CoV-2 infections is unlikely to be homogeneous across all communities and regions. We observed higher seroprevalence in the urban site than in the rural site for the respective survey rounds. The urban informal settlement environments, such as Kibera in Nairobi, may have been disproportionately affected due to overcrowding, water, sanitation, and hygiene (WASH) infrastructure constraints, and socio-economic challenges that make implementation of COVID-19 public health mitigation measures difficult. Additionally, proximity to the capital city, the main hub for local and international travel, may have led to early introduction and continued population exposure to SARS-CoV-2 especially with the more transmissible variants. A serosurvey in July 2020 in Mumbai, India found the seroprevalence among informal settlement residents to be nearly 3.6 times that of formal settlement residents ^17^, which is in line with our findings. The prevalence of anti-SARS-CoV-2 antibodies increased in the urban population from 56% in March 2021 to 64% by June 2021. A previous survey in Kibera in December had reported seroprevalence of 43% ^10^. Most (87%) of the households had been exposed to SARS-CoV-2 by June 2021 in Kibera. Despite the high seroprevalence (albeit with low COVID-19 vaccination coverage) in the urban informal settlement, we observed a high-level of transmission in the fourth (predominated by Delta variant) and fifth (Omicron variant) waves of SARS-CoV-2 circulation, with peak weekly PCR-positivity of 87% in December 2021. In rural western Kenya, the seroprevalence before the second wave was low at 26% but almost doubled after the Delta variant circulation to 49%. The transmission of the SARS-CoV-2 in the third wave was also high in Asembo (peak PCR positivity of 50% in December 2021). The continued transmission in both sites highlights the importance of prioritizing additional mitigation measures, including COVID-19 vaccine distribution in these low socio-economic settings.

An individual’s age and household size were the main predictors for exposure to SARS-CoV-2 infection across the two sites. The lower seroprevalence in children under 10 years of age could be attributable to milder infections in this group, which could be associated with lower antibody titres. Furthermore, adults in both populations would have increased exposure from daily travel to work and/or at workplaces, partly explaining the increased seroprevalences in the adult age groups. Given that the vast majority (87%) of the households had at least one person infected by time of the second serosurvey, the age differences were less obvious, and we expect these differences would dissipate with the recent extensive community circulation of the more transmissible variants such as the Omicron. Close contacts and crowding within the household could explain why the number of household members was an important risk factor for previous exposure. Persons sharing living spaces have increased risk of transmission for respiratory infections within the household, and large households would also have a greater risk of importing infections from outside of the household ^32^.

Though our follow-up of the study participants was limited to an interval of about three months, we observed potential sero-reversions among the previously seropositive individuals. Unlike in Asembo where there were no differences in median ages of those who sero-reverted and the rest, the sero-revertors were older compared to those who maintained their serostatus or seroconverted in Kibera. These observations point to the possible waning of mainly infection-induced immunity, but longer-term follow-up of the cohort would be required to delineate the antibody dynamics and kinetics by age. A longitudinal follow-up of recovered COVID-19 patients showed persistence of neutralizing antibody response beyond six months, though great variations in duration of neutralizing antibody responses (and T-cell responses) that were specific to individual characteristics were observed ^33^. Post-Omicron studies would be required to show the longevity of the antibodies and duration of protective immunity against infection and, disease especially in the context of infection, vaccine-induced or hybrid immunity ^34, 35^. Recent studies have shown that the risk of reinfection has been lower in previous Variants of Concern (VOCs) compared with the immune-escaping Omicron variant in previously infected as well as vaccinated individuals ^36, 37^. This demonstrates that the presence of antibodies is not a perfect correlate of the level of protection against infection suggesting interpretation of seropositivity status may be challenging. Nonetheless, the presence of SARS-CoV-2 antibodies remains indicative of protection against severe disease and population-based seroprevalence studies would primarily provide reliable estimates of the level of exposure to the infection ^25, 38^.

The study is not without some limitations, and some have been highlighted in our early publications ^10, 31^. First, selection bias could have occurred as not all individuals from the randomly selected households were enrolled as expressed by age and sex variations in the probabilities for inclusion of the PBIDS population ( Figure 3). However, we weighted our population level estimates to account for these variations as described previously ^10^. Second, we did not adjust the reported seroprevalence for assay performance. Diagnostic performance estimates from local or similar populations were missing to provide a meaningful interpretation. Third, some asymptomatic individuals may not seroconvert, some individuals may have been tested prior to seroconversion, and others may have antibodies that had waned by the time of blood collection, so the data in this study may underestimate the true evidence of SARS-CoV-2 infections. Our assay tested for anti-spike IgG antibodies, which show better persistence in serum and may mitigate this problem. Fourth, seropositivity was not confirmed by a neutralization or a secondary assay and potential cross-reactivity with endemic coronaviruses could not be ruled out. Finally, our estimates arise from two underserved populations in Kenya, urban poor and rural communities with limited access to health care and may have limited generalizability in generating national estimates.

In conclusion, we observed continued transmission of SARS-CoV-2 in two diverse populations with high infection exposure and low vaccination uptake in Kenya. The implementation of mitigation measures—such as case identification and isolation, contact tracing and quarantine, and social distancing and uptake of COVID-19 vaccines may have been very challenging in these populations. Despite the high SARS-CoV-2 seroprevalence in urban informal settlements, more transmissible and/or immune escaping variants of concern continued to spread in urban informal settlements. It might be, however, important to prioritize additional mitigation measures, such as COVID-19 vaccine distribution, in these crowded and low socioeconomic settings.

## Data Availability

As the dataset contains potentially identifying information on participants, it is stored under restricted access. For more detailed information beyond the metadata and documentation provided, there is a process of managed access requiring the submission of a request for consideration by our Data Governance Committee. Please contact the Data Governance Committee via this email address: GBigogo@kemri.go.ke.
