# Supplementary material for: Heterogenous transmission and seroprevalence of SARS-CoV-2 in two demographically diverse populations with low vaccination uptake in Kenya, March and June 2021
Source: Gates Open Res. 2023 Oct 9;7:101. Originally published 2023 Jul 4. [Version 2] doi: 10.12688/gatesopenres.14684.2 (PMC10661969; doi:10.12688/gatesopenres.14684.2)
Supplement: Peer review history

# Reviewer report: Reviewer response for version 2

**DOI:** 10.21956/gatesopenres.16369.r35243

**Published:** 2024-01-25

**Authors:** Stefania Paduano

**Affiliations:** 1 University of Modena and Reggio Emilia, Modena, Italy

No further comments.

Is the work clearly and accurately presented and does it cite the current literature?

Partly

If applicable, is the statistical analysis and its interpretation appropriate?

Partly

Are all the source data underlying the results available to ensure full reproducibility?

Yes

Is the study design appropriate and is the work technically sound?

Partly

Are the conclusions drawn adequately supported by the results?

Yes

Are sufficient details of methods and analysis provided to allow replication by others?

Partly

Reviewer Expertise:

Hygiene, Infectious dieseas epidemiology, Public Health

I confirm that I have read this submission and believe that I have an appropriate level of expertise to confirm that it is of an acceptable scientific standard.

# Reviewer report: Reviewer response for version 2

**DOI:** 10.21956/gatesopenres.16369.r35244

**Published:** 2023-11-20

**Authors:** Sri Masyeni, I Made Wisnu Wardhana

**Affiliations:** 1 Universitas Warmadewa/Sanjiwani Hospital, Bali, Indonesia; 2 University of Warmadewa, Denpasar, Bali, Indonesia

No further comments to make.

Is the work clearly and accurately presented and does it cite the current literature?

Yes

If applicable, is the statistical analysis and its interpretation appropriate?

Partly

Are all the source data underlying the results available to ensure full reproducibility?

Partly

Is the study design appropriate and is the work technically sound?

Yes

Are the conclusions drawn adequately supported by the results?

Yes

Are sufficient details of methods and analysis provided to allow replication by others?

Yes

Reviewer Expertise:

Tropical and Infectious Disease

We confirm that we have read this submission and believe that we have an appropriate level of expertise to confirm that it is of an acceptable scientific standard.

# Reviewer report: Reviewer response for version 1

**DOI:** 10.21956/gatesopenres.16008.r34082

**Published:** 2023-08-22

**Authors:** Stefania Paduano

**Affiliations:** 1 University of Modena and Reggio Emilia, Modena, Italy

The authors (AA) aim to evaluate the seroprevalence of SARS-CoV-2 in two well-characterized populations in Kenya at two-time points.

 This is an engaging article useful to increase our knowledge of the issue. Addressing all the issues below reported could make this manuscript eligible for indexing.

**Title:**

- AA should drop March and June 2021 and insert that the survey was performed in two time points in 2021.

**Abstract:**

- AA should be careful to correctly report the two-time points: in the methods, June 2021 is written while in the results June (Kibera) and July (Asembo) 2021.
- In the conclusions, AA report a rapid increase in the first 16 months, but I would suggest expressing their conclusions in a different way since the authors did not record the seroprevalence trend over the 16 months but only in two-time points.

**Introduction:**

- AA could add some references such as: Ferrari *et al.* (2023 ^1^).

**Methods:**

- Figure 1: Improve resolution of the figure.
- Explain better in the text the meaning of Figure 3.

**Results:**

- Figure 4: does the number of tests refer to RT-PCR tests? Moreover, the serosurvey periods do not correspond to those indicated in the methods. Clarify it.
- Were 3 serological tests carried out in Kibera?
- Regarding those who had the vaccine but were not seropositive, was it considered how much time had passed since the vaccine dose and whether or not they had previously contracted the infection?
- AA should take into consideration the type of work of the included subjects considering results reported by some authors such as Modenese *et al.* (2021 ^2^).
- Have AA considered the grade of the schools attended by the minors?

**Discussion:**

- AA should report and consider the preventive measures implemented in the first sixteen months in Asembo and Kibera in order to reduce SARS-CoV-2 transmission.
- The following studies could be considered for discussion:
  - Paduano *et al. *(2022 ^3^).
  - Berselli *et al. *(2022 ^4^).
  - Paduano *et al. *(2021 ^5^).

Is the work clearly and accurately presented and does it cite the current literature?

Partly

If applicable, is the statistical analysis and its interpretation appropriate?

Partly

Are all the source data underlying the results available to ensure full reproducibility?

Yes

Is the study design appropriate and is the work technically sound?

Partly

Are the conclusions drawn adequately supported by the results?

Yes

Are sufficient details of methods and analysis provided to allow replication by others?

Partly

Reviewer Expertise:

Hygiene, Infectious dieseas epidemiology, Public Health

I confirm that I have read this submission and believe that I have an appropriate level of expertise to confirm that it is of an acceptable scientific standard, however I have significant reservations, as outlined above.

## References

[1] : Long-Term SARS-CoV-2 Antibody Seroprevalence in Blood Donors, Italy. *Emerg Infect Dis* .2023;29(7) : 10.3201/eid2907.221745 1479-1481 10.3201/eid2907.221745 37253276 PMC10310387

[2] : Frequency of Anti-SARS-CoV-2 Antibodies in Various Occupational Sectors in an Industrialized Area of Northern Italy from May to October 2020. *Int J Environ Res Public Health* .2021;18(15) : 10.3390/ijerph18157948 10.3390/ijerph18157948 34360241 PMC8345498

[3] : Seroprevalence Survey of Anti-SARS-CoV-2 Antibodies in a Population of Emilia-Romagna Region, Northern Italy. *Int J Environ Res Public Health* .2022;19(13) : 10.3390/ijerph19137882 10.3390/ijerph19137882 35805539 PMC9266015

[4] : Seroprevalence of anti-SARS-CoV-2 antibodies in the Northern Italy population before the COVID-19 second wave. *Int J Occup Med Environ Health* .2022;35(1) : 10.13075/ijomeh.1896.01826 63-74 10.13075/ijomeh.1896.01826 34524275 PMC10464740

[5] : Characteristics and risk factors of isolated and quarantined children and adolescents during the first wave of SARS-CoV-2 pandemic: A cross-sectional study in Modena, Northern Italy. *Acta Biomed* .2021;92(S6) : 10.23750/abm.v92iS6.12225 e2021449 10.23750/abm.v92iS6.12225 34739471 PMC8850999

# Response

**Published:** 2023-09-27

**Authors:** Patrick Munywoki

**Affiliations:** CDC Kenya, Kenya

1. Title:

- AA should drop March and June 2021 and insert that the survey was performed in two time points in 2021.

*Thank for your kind review of the manuscript. While we respect your suggestion, we strongly feel including the approximate surveillance month would immediately help our audience to capture the serosurvey time points in 2021. the level of SARS-CoV-2 exposure as measured by serological assay varied over time and we argue that specifying the exact timepoint is important. Furthermore, the interpretation of the population-level seroprevalence estimates is dependent on the timing of the serosurvey in relation to SARS-CoV-2 circulation.*

 2. Abstract:

- AA should be careful to correctly report the two-time points: in the methods, June 2021 is written while in the results June (Kibera) and July (Asembo) 2021.

 
*We have reviewed the abstract and the results section for consistency in reporting the survey months for the two sites. Thank you.*

- In the conclusions, AA report a rapid increase in the first 16 months, but I would suggest expressing their conclusions in a different way since the authors did not record the seroprevalence trend over the 16 months but only in two-time points.

*The SARS-CoV-2 was introduced in these population in early 2020, and trends shown in three timepoints in Kibera and two timepoints in Asembo show a marked increase in seropositivity in the first 16 months. We have made a minor edit in the conclusion to reflect this.*

 3. Introduction:

- AA could add some references such as: Ferrari et al. (2023 1).

*Thank you for the suggestion. The reference has been added.*

 4. Methods:

- Figure 1: Improve resolution of the figure.

*A new improved figure has been submitted. Thank you.*


*  *

- Explain better in the text the meaning of Figure 3.

*Figure 3 shows the participant's probability of being selected in each site in reference to the overall PBIDS population-size stratified by age and sex at the two survey timepoints. This has been clarified in the article.*

 5. Results:

- Figure 4: does the number of tests refer to RT-PCR tests? Moreover, the serosurvey periods do not correspond to those indicated in the methods. Clarify it.

*Thank you for this correction. We have revised Figure 4 with the correct weighted seroprevalence values and refining the serosurvey periods. The number of tests refers to PCR tests and this now explicitly stated in the revised figure.*


*  *

- Were 3 serological tests carried out in Kibera?

*Yes, the additional serosurvey was carried in December 2020 and data has been published elsewhere and indicated in this article to provide context for interpretating the 2021 results.*


*  *

- Regarding those who had the vaccine but were not seropositive, was it considered how much time had passed since the vaccine dose and whether or not they had previously contracted the infection?

*Thank you pointing this out. We added results on the duration since the COVID-19 vaccine in the results section. "The median duration (IQR) for those seronegative after receiving COVID-19 vaccine in both sites (n=5) was 42 (4-72) days compared to 63.5 (3-100) days for those seropositive, though not statistically significant - Wilcoxon rank-sum test p-value=0.5490." None of the five participants had reported being diagnosed with COVID-19 in preceeding period.*


*  *

- AA should take into consideration the type of work of the included subjects considering results reported by some authors such as Modenese et al. (2021 2).

*Majority of our participants were either self-employed or in informal employment and data on the type of work was not systematically collected. An attempt to apply the International Standard Industrial Classification of All Economic Activities as reported in the referenced article resulted to a lot of individuals with missing values.*


* *

- Have AA considered the grade of the schools attended by the minors?

*This was considered in the preliminary analyses. There were no statistically significant differences by grades after accounting for age differences hence why not included in the article.*

 6. Discussion:

- AA should report and consider the preventive measures implemented in the first sixteen months in Asembo and Kibera in order to reduce SARS-CoV-2 transmission.

*These were generally described in the introduction and discussion to help in interpretation of the results. The level of adherence to the non-pharmaceutical interventions was variable and not tracked to allow detailed listing.*


*  *

- The following studies could be considered for discussion:

Paduano et al. (2022 3).

 Berselli et al. (2022 4).

 Paduano et al. (2021 5).

*Thank you for the suggestions. While the articles report relevant seroprevalence studies in Italy, we opted to reference studies from sub-Saharan Africa and similar settings.*

# Reviewer report: Reviewer response for version 1

**DOI:** 10.21956/gatesopenres.16008.r34074

**Published:** 2023-08-02

**Authors:** Sri Masyeni, I Made Wisnu Wardhana

**Affiliations:** 1 Universitas Warmadewa/Sanjiwani Hospital, Bali, Indonesia; 2 University of Warmadewa, Denpasar, Bali, Indonesia

The article's findings highlight how SARS-CoV-2 spread swiftly without proper preventive measures in two distinct populations: dense urban populations and sparse rural populations. More than 70%  of the cited literature was published within 5 years of this study. The study design was acceptable, but the Author need to clarify the manner in which 900 individuals were determined as the total number of participants for each population of the study. In addition, rather than a descriptive study, an analytical study demonstrating factors that correspond with differences between these two populations would be more scientifically appealing. The Author went into great detail on the study technique and analysis. The statistical analysis was presented in an informative table and figure, but its interpretation was unclear. To reduce repetition, the author should explain the outcome in their own words rather than typing the data from the table again. To safeguard the subject's confidentiality, the source data was limited and only available through a request. The conclusion provided a summary of the study and was based on the results and discussion.

Is the work clearly and accurately presented and does it cite the current literature?

Yes

If applicable, is the statistical analysis and its interpretation appropriate?

Partly

Are all the source data underlying the results available to ensure full reproducibility?

Partly

Is the study design appropriate and is the work technically sound?

Yes

Are the conclusions drawn adequately supported by the results?

Yes

Are sufficient details of methods and analysis provided to allow replication by others?

Yes

Reviewer Expertise:

Tropical and Infectious Disease

We confirm that we have read this submission and believe that we have an appropriate level of expertise to confirm that it is of an acceptable scientific standard, however we have significant reservations, as outlined above.

# Response

**Published:** 2023-09-27

**Authors:** Patrick Munywoki

**Affiliations:** CDC Kenya, Kenya

*Thank you for your detailed review. Below we provide a point-to-point response to the comments raised:*

- *The study participants were recruited from randomly selected households in each site as shown in Figure 2. This has been clarified in methods under household-based seroprevalence surveys section.*
- *The manuscript has both descriptive and analytical components. Table 5 shows the risk factors for individual seropositivity by site and survey round. A separate analytical analysis of the PCR-data from the health facility COVID-19 surveillance is ongoing and will assess the determinants of SARS-CoV-2 infection in the two populations.  *
- *We have reviewed the results description in the methods for clarity and consistency in interpretation. *

# Reviewer report: Reviewer response for version 1

**DOI:** 10.21956/gatesopenres.16008.r34075

**Published:** 2023-07-27

**Authors:** Raquel A Binder

**Affiliations:** 1 University of Massachusetts Chan Medical School, Worcester, USA

This study leverages two longitudinal cohorts in Kenya (one rural - Asembo, and one urban - Kibera) to assess the SARS-CoV-2 prevalence via a commercial serological assay in 2021 and the associated risk factors for positivity. This is a thorough and extensive study with interesting results. It is a very well-written report with valuable information on seropositivity across ages, sex, and rural-urban settings among other assessed variables, and vaccine distribution/uptake. 

 The source data (i.e., raw data - serological results linked to demographics and other captured variables by site etc.) are not available (I do not see it on this portal). Hence, not all the source data underlying the results are currently available. 

 I chose 'approved with reservations' due to the below comments which can be easily addressed. Otherwise, it is a rigorous study that is ready for indexing.

Please see comments below:

1. There are no details on the thresholds and potential cross-reactivity with endemic coronaviruses with respect to the SCoV-2 Detect ELISA. This may be inducing errors and should be mentioned in limitations. 
2. PCR results are included in Fig. 4 and mentioned in the methods but are not mentioned in Fig. 2. Are the PCR results and serology linked for the individuals? A risk factor analysis (as was done for serology) for PCR positivity would be valuable. 
3. Figure 4: Top section (Asembo), has the crude (not the overall weighted) seroprevalence listed, according to Table 3.
4. Figure 4: Bottom section (Kibera), has three seroprevalence points listed but only two (last two) are discussed in the paper. Where did the Nov seroprevalence come from?
5. Results. “Individual seroprevalence of SARS-CoV-2 of SARS-CoV-2 antibodies” section, third paragraph. The education analysis seems to have been done with the inclusion of children that could not possibly have undergone schooling yet. Hence, the educational analysis and discussion should be adjusted to either exclude children or adapt the categories. 
6. Discussion. The fourth paragraph discusses clinical outcomes among the population. Were clinical outcomes captured in the survey? No mention in methods or more detailed discussion in manuscript otherwise.  
7. Discussion. Seroreversion (comparing the two measured time points, ~2-4 months apart) is discussed but the time point of infection is not identified. Hence, the time since infection is the important timeframe when analyzing antibody decay. The time of infection likely varied for each participant at each time point (R1 and R2) and can therefore not be compared.
8. The map (Fig.1) can be cropped to encompass a fraction of the size and cover the sites of interest.

Minor points:

1. Introduction. Second paragraph, second sentence: needs reference. (Most infected persons generate detectable antibodies that persist for up to a year.)
2. Methods. Second paragraph, first sentence: check word “thrice”?
3. Discussion. First paragraph, first sentence: “Leveraging well-characterized populations in two diverse geographical locations…” (delete second word "on")
4. Discussion. First paragraph, first sentence: this sentence needs reference “For instance, the first COVID-19 case was not detected until September 2020 in the rural site and the Delta…”  
5. Discussion. Second paragraph: “A serosurvey in July 2020 in Mumbai, India found the seroprevalence among informal settlement residents to be nearly 3.6 times that of formal settlement residents, which is in line with our findings.” (delete non-informal).

Is the work clearly and accurately presented and does it cite the current literature?

Yes

If applicable, is the statistical analysis and its interpretation appropriate?

Yes

Are all the source data underlying the results available to ensure full reproducibility?

Partly

Is the study design appropriate and is the work technically sound?

Yes

Are the conclusions drawn adequately supported by the results?

Yes

Are sufficient details of methods and analysis provided to allow replication by others?

Partly

Reviewer Expertise:

Infectious disease epidemiology and serology, with focus on COVID-19.

I confirm that I have read this submission and believe that I have an appropriate level of expertise to confirm that it is of an acceptable scientific standard, however I have significant reservations, as outlined above.

# Response

**Published:** 2023-09-27

**Authors:** Patrick Munywoki

**Affiliations:** CDC Kenya, Kenya

This study leverages two longitudinal cohorts in Kenya (one rural - Asembo, and one urban - Kibera) to assess the SARS-CoV-2 prevalence via a commercial serological assay in 2021 and the associated risk factors for positivity. This is a thorough and extensive study with interesting results. It is a very well-written report with valuable information on seropositivity across ages, sex, and rural-urban settings among other assessed variables, and vaccine distribution/uptake. 

 The source data (i.e., raw data - serological results linked to demographics and other captured variables by site etc.) are not available (I do not see it on this portal). Hence, not all the source data underlying the results are currently available. 

 I chose 'approved with reservations' due to the below comments which can be easily addressed. Otherwise, it is a rigorous study that is ready for indexing.

*Thank you for a thorough review of our manuscript. On the data availability, the data is on restricted access for it contains person identifiers and is subject to further analysis as new rounds of serosurvey are planned. The data can be shared following request to the Project’s Data Governance Committee at Centre for Global Health, Kenya Medical Research Institute.*

 Please see comments below:

 1. There are no details on the thresholds and potential cross-reactivity with endemic coronaviruses with respect to the SCoV-2 Detect ELISA. This may be inducing errors and should be mentioned in limitations. 

*A sentence has been added regarding the limitations associated with possible cross-reactivity of the assay used. Thank you for the suggestion.*

 2. PCR results are included in Fig. 4 and mentioned in the methods but are not mentioned in Fig. 2. Are the PCR results and serology linked for the individuals? A risk factor analysis (as was done for serology) for PCR positivity would be valuable. 

*Figure 2 is showing the sampling flow for the seroprevalence surveys, the focus for this paper. The PCR tests were from patients seeking medical care at the centrally located health facilities in each site. The PCR results in Figure 4 are shown to provide local context on SARS-CoV-2 circulation in the two surveillance sites. The temporal SARS-CoV-2 infection detection patterns help in interpretation of the seroprevalence estimates. A separate analysis is ongoing looking at the risk factors for SARS-CoV-2 infection and their outcomes stratified by the COVID-19 waves in the two surveillance sites.*

**

 3. Figure 4: Top section (Asembo), has the crude (not the overall weighted) seroprevalence listed, according to Table 3.

*Thank you for picking that error. We have revised figure 4 to reflect overall weighted seroprevalence and corrected the survey period lines.*

**

 4. Figure 4: Bottom section (Kibera), has three seroprevalence points listed but only two (last two) are discussed in the paper. Where did the Nov seroprevalence come from?

*In the November/December 2020, we had a serosurvey conducted only in Kibera and we have added the values here for reference. The December 2020 serosurvey in Kibera has been published and is described and referenced in the article.*

**

 5. Results. “Individual seroprevalence of SARS-CoV-2 of SARS-CoV-2 antibodies” section, third paragraph. The education analysis seems to have been done with the inclusion of children that could not possibly have undergone schooling yet. Hence, the educational analysis and discussion should be adjusted to either exclude children or adapt the categories. 

*Education level is not independently associated individual seropositivity. An analysis with the suggested reclassification of the education variable has not yielded any statistically significant differences to affect interpretation of the results*. 

 6. Discussion. The fourth paragraph discusses clinical outcomes among the population. Were clinical outcomes captured in the survey? No mention in methods or more detailed discussion in manuscript otherwise.  

*Clinical outcomes were not collected as part of this cross-sectional survey.*

 7. Discussion. Seroreversion (comparing the two measured time points, ~2-4 months apart) is discussed but the time point of infection is not identified. Hence, the time since infection is the important timeframe when analyzing antibody decay. The time of infection likely varied for each participant at each time point (R1 and R2) and can therefore not be compared.

*While we acknowledge the limitations of our data in looking at antibody decay without data on the date of infection, it would be informative to show the antibody persistence (or lack of) in the two timepoints of this study. Additionally, possibility of reinfections also makes the interpretation of our data also more challenging and limiting.*

**

 8. The map (Fig.1) can be cropped to encompass a fraction of the size and cover the sites of interest.

*Thank you. We have cropped the map accordingly.*

 Minor points:

 9. Introduction. Second paragraph, second sentence: needs reference. (Most infected persons generate detectable antibodies that persist for up to a year.)

*A reference has been added. Thank you.*

**

 10. Methods. Second paragraph, first sentence: check word “thrice”?

*We have changed it to ‘three times’ for clarity. Thank you.*

**

 11. Discussion. First paragraph, first sentence: “Leveraging well-characterized populations in two diverse geographical locations…” (delete second word "on")

*Revised to read, ‘Leveraging on well-characterized populations in two diverse geographical locations.*

**

 12. Discussion. First paragraph, first sentence: this sentence needs reference “For instance, the first COVID-19 case was not detected until September 2020 in the rural site and the Delta…”  

*The sentence is based on interpretation of the actual data presented in figure 4.*

 13. Discussion. Second paragraph: “A serosurvey in July 2020 in Mumbai, India found the seroprevalence among informal settlement residents to be nearly 3.6 times that of formal settlement residents, which is in line with our findings.” (delete non-informal).

*Thank you for the corrections. We have replaced the word ‘non-informal’ with ‘formal’.*
